# Supplementary material for: Serum Metabolomic and Lipidomic Profiling Reveals Novel Biomarkers of Efficacy for Benfotiamine in Alzheimer’s Disease
Source: Int J Mol Sci. 2021 Dec 7;22(24):13188. doi: 10.3390/ijms222413188 (PMC8709126; doi:10.3390/ijms222413188)
Supplement: Supplementary file 1 [file ijms-22-13188-s001.zip › Supplementary Word and Figures document FINAL.pdf]

SUPPLEMENTARY DOCUMENT

Serum metabolomic and lipidomic profiling reveals novel biomarkers of efficacy for Benfotiamine in Alzheimer's disease

Ruchika Bhawal[1], Qin Fu[1], Elizabeth T. Anderson[1], Gary E. Gibson[2], Sheng Zhang[1*]

[1]Proteomics and Metabolomics Facility, Institute of Biotechnology, Cornell University, Ithaca, NY, 14853 USA; [2]Brain and Mind Research Institute, Weill Cornell Medicine; Burke Neurological Institute, White Plains, NY, 10605 USA.

*Corresponding author: Sheng Zhang, E-mail: sz14@cornell.edu

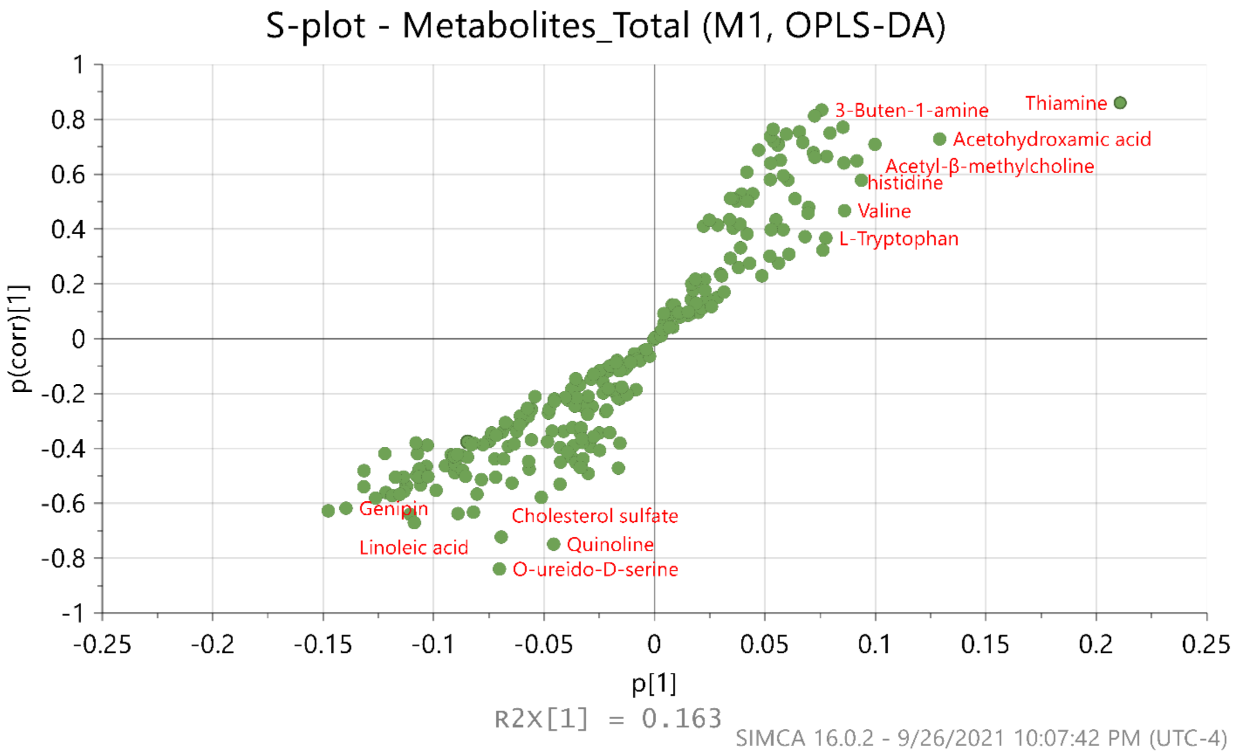

Fig. S1 – S-plot generated from OPLS-DA model for metabolites identified in placebo and Benfotiamine treated samples. In an S-plot, the x variable is the relative magnitude of a variable, and the y variable is the variable confidence/reliability. So, data points falling in the upper right or lower left corners of the plot represent those features that are least likely to be the result of spurious correlations. Peaks with low magnitude/intensity falling in the center of the plot near 0 are close to the noise level and exhibit high risks for spurious correlations.

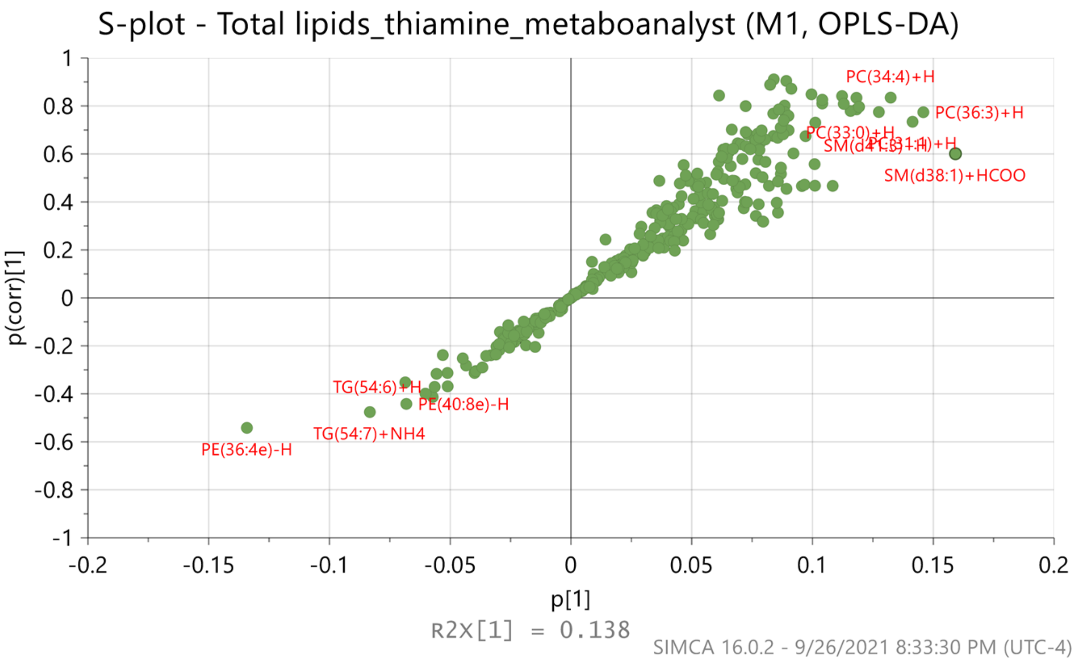

Fig. S2 – S-plot generated from OPLS-DA model for lipid ions identified in placebo and Benfotiamine treated samples. In an S-plot, the x variable is the relative magnitude of a variable, and the y variable is the variable confidence/reliability. So, data points falling in the upper right or lower left corners of the plot represent those features that are least likely to be the result of spurious correlations. Peaks with low magnitude/intensity falling in the center of the plot near 0 are close to the noise level and exhibit high risks for spurious correlations.

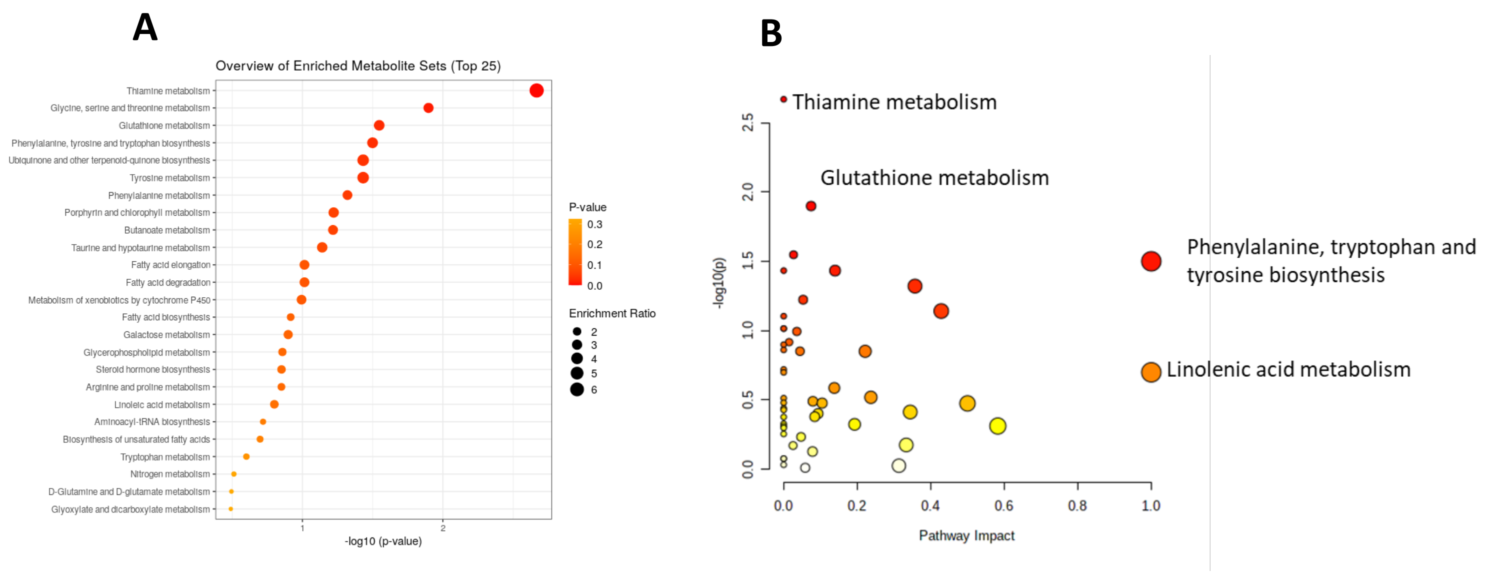

Fig. S3: Enrichment analysis for metabolite dataset based on differentially accumulated metabolites identified in serum (A); and its pathway impact analysis by MetaboAnalyst 5.0 (B). Four major relevant pathways were found including (a) thiamine metabolism; (b) Phenylalanine, tyrosine and tryptophan biosynthesis; (c) Glutathione metabolism and (d) Linoleic acid metabolism. Color gradient and circle size indicate the significance of the pathway ranked by p-value (yellow: higher p-values and red: lower p-values) and pathway impact score (the larger the circle the higher the impact score), respectively. Pathway enrichment analysis computes a single P value for each metabolic pathway (a group of functional-associated metabolites), as opposed to the t test, which calculates statistical significance of the difference between individual metabolites, hence some of the predication may not be in line with BFT treatment. A “pathway impact score” was then computed as the sum of the importance measures of identified metabolites divided by the total sum of the importance measures of all the identified and unidentified metabolites in the pathway.

Supplementary Table S1: Summary table for all the number of metabolites detected and identified after filtering in metabolomics workflow with various levels of confidence.

Supplementary Table S2: Summary table for all the number of lipid species detected and identified in lipidomics workflow with various levels of confidence.
